# Cannulated Cancellous Screw Fixation for Symptomatic Os Subfibulare: A Case Report

**DOI:** 10.7759/cureus.77755

**Published:** 2025-01-21

**Authors:** Yuko Yagi, Takeomi Nakamura

**Affiliations:** 1 Department of Orthopedics, Tokyo Metropolitan Hiroo Hospital, Tokyo, JPN

**Keywords:** accessory ossicle, anterior talofibular ligament, cannulated cancellous screw fixation, chronic ankle instability, chronic ankle pain, os subfibulare

## Abstract

Os subfibulare, a rare accessory ossicle located at the distal fibula, is hypothesized to result from the failure of fusion of a secondary ossification center or chronic avulsion fractures. While often asymptomatic, symptomatic cases associated with pain and ankle instability may require surgical intervention when conservative management fails. This report describes the case of a 16-year-old female who was successfully treated with cannulated cancellous screw fixation, achieving complete symptom resolution, restoring ankle stability, and significant functional improvement. This case highlights the importance of selecting optimal surgical strategies for large fragments to restore joint stability and improve outcomes. Given the short-term follow-up of this study, further research is necessary to evaluate long-term efficacy and refine treatment protocols.

## Introduction

Os subfibulare is a rare accessory ossicle at the distal fibula, arising from incomplete fusion of a secondary ossification center or repetitive trauma, such as avulsion fractures involving the anterior talofibular ligament (ATFL) [1-3]. Its prevalence ranges from 1% to 6.7% in the general population, increasing to 10% to 38.5% in individuals with chronic ankle instability [4-7]. While asymptomatic in most cases, symptomatic presentations can include chronic pain, functional limitations, and persistent instability.

Conservative management, including physical therapy and activity modification, is typically the first-line approach. However, surgical intervention may be required in cases refractory to these measures [1]. Surgical options include fragment excision, ligament reconstruction, and fixation, but a standardized treatment algorithm has not yet been established due to limited evidence [8]. Furthermore, the lack of comprehensive studies on fixation techniques emphasizes the need for further research to optimize treatment strategies for symptomatic os subfibulare.

This article was previously presented as a poster at the 49th Annual Meeting of the Japanese Society for Surgery of the Foot on November 7, 2024.

## Case presentation

A 16-year-old female with no significant medical history presented with persistent right ankle pain and instability following a fall down stairs. Initially diagnosed with a distal fibula fracture, she underwent conservative management at a local clinic. However, her symptoms persisted, including activity-limiting pain, prompting her referral to our institution 10 months post injury.

**Figure 1:**
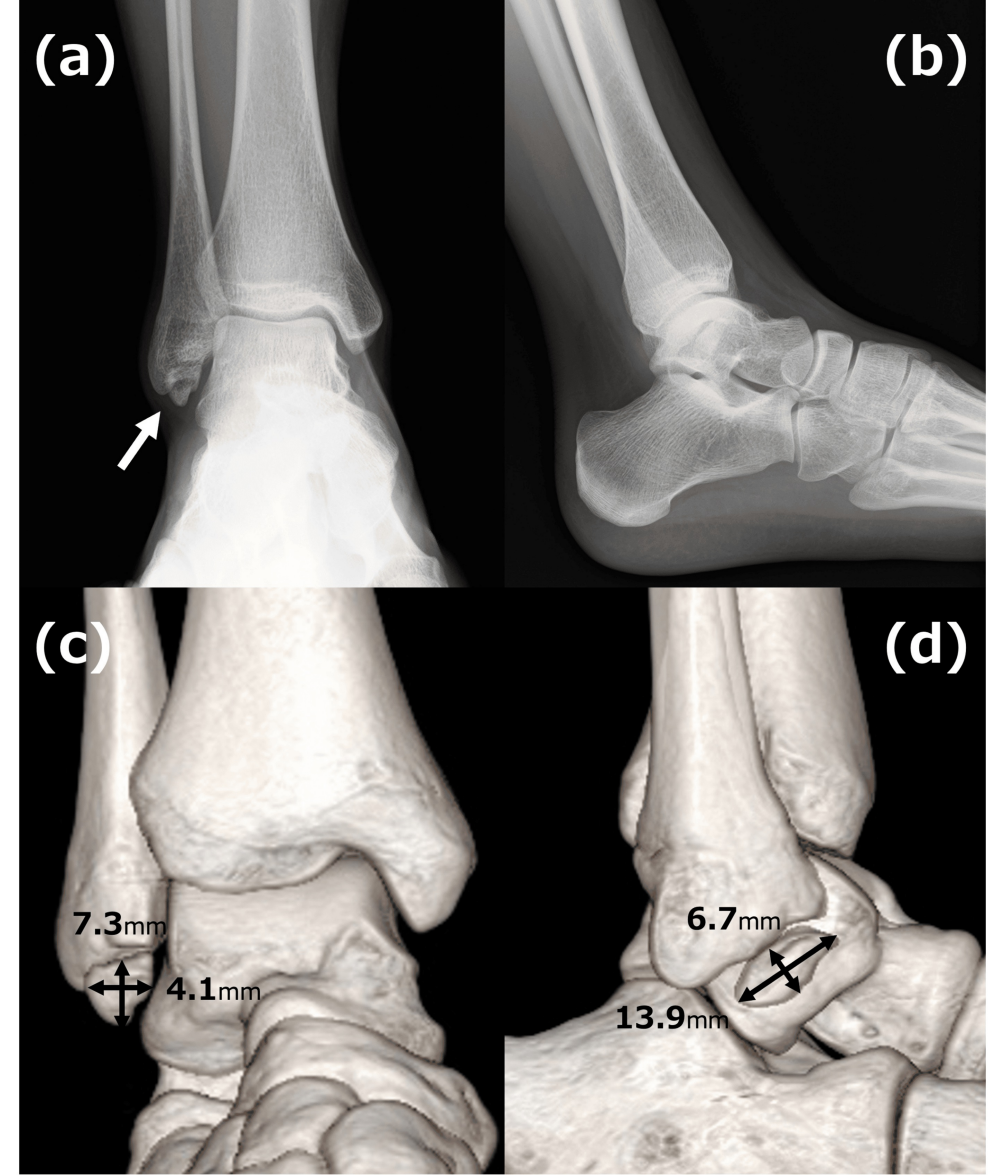
Radiographic and 3D CT imaging of the ankle. Plain radiographs: Anteroposterior view (a) and lateral view (b). An arrow identifies the os subfibulare. 3D reconstructed CT images: Anterior view (c) and lateral view (d). The os subfibulare measures 14 mm in maximum diameter.

Clinical examination revealed tenderness over the distal fibula, pain exacerbated by activity, and a positive anterior drawer test. Radiographic imaging, including plain X-rays and computed tomography (CT) scans, confirmed a symptomatic os subfibulare measuring 14 mm in its largest dimension (Figure 1).

**Figure 2:**
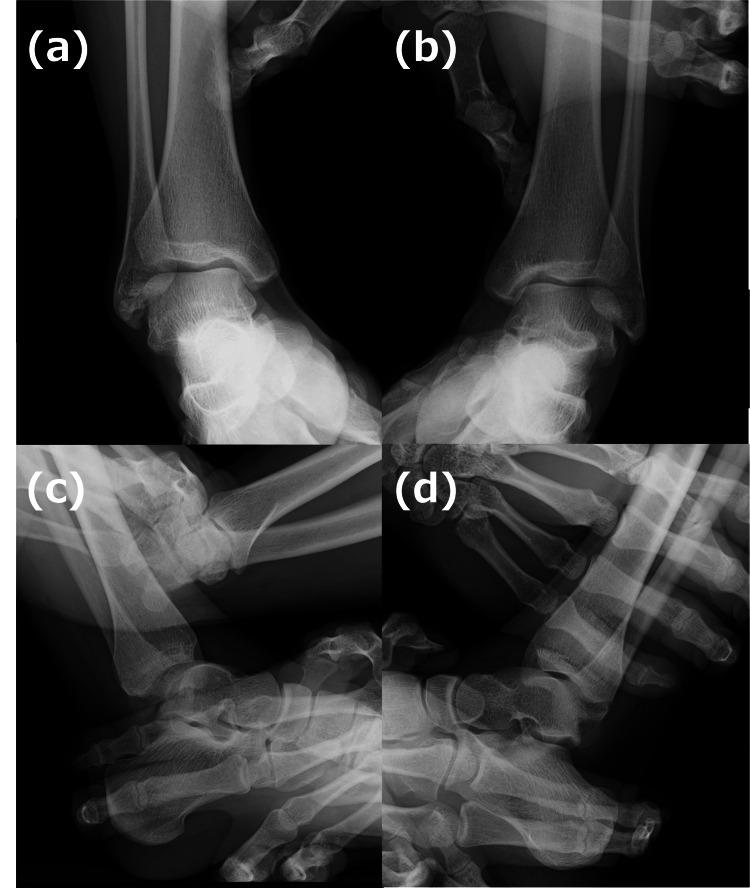
Stress test results of the ankle. Inversion stress test: A comparison of the affected side (a) and unaffected side (b) shows excessive talar tilt on the affected side. Anterior drawer test: A comparison of the affected side (c) and unaffected side (d) demonstrates increased anterior talar translation on the affected side.

Stress radiographs demonstrated increased talar tilt and anterior talar translation, indicative of chronic instability (Figure 2) [9].

Surgical intervention was deemed necessary due to the patient’s persistent symptoms and functional impairment. Intraoperative findings included pseudarthrosis at the os subfibulare with fibrocartilaginous changes and sclerosis. The ATFL was intact and attached to the fragment, maintaining physiological tension. The pseudarthrosis site was debrided, and fixation was performed using two 2.2 mm cannulated cancellous screws (CCSs). Post-fixation, stability was confirmed intraoperatively with a negative anterior drawer test.

**Figure 3:**
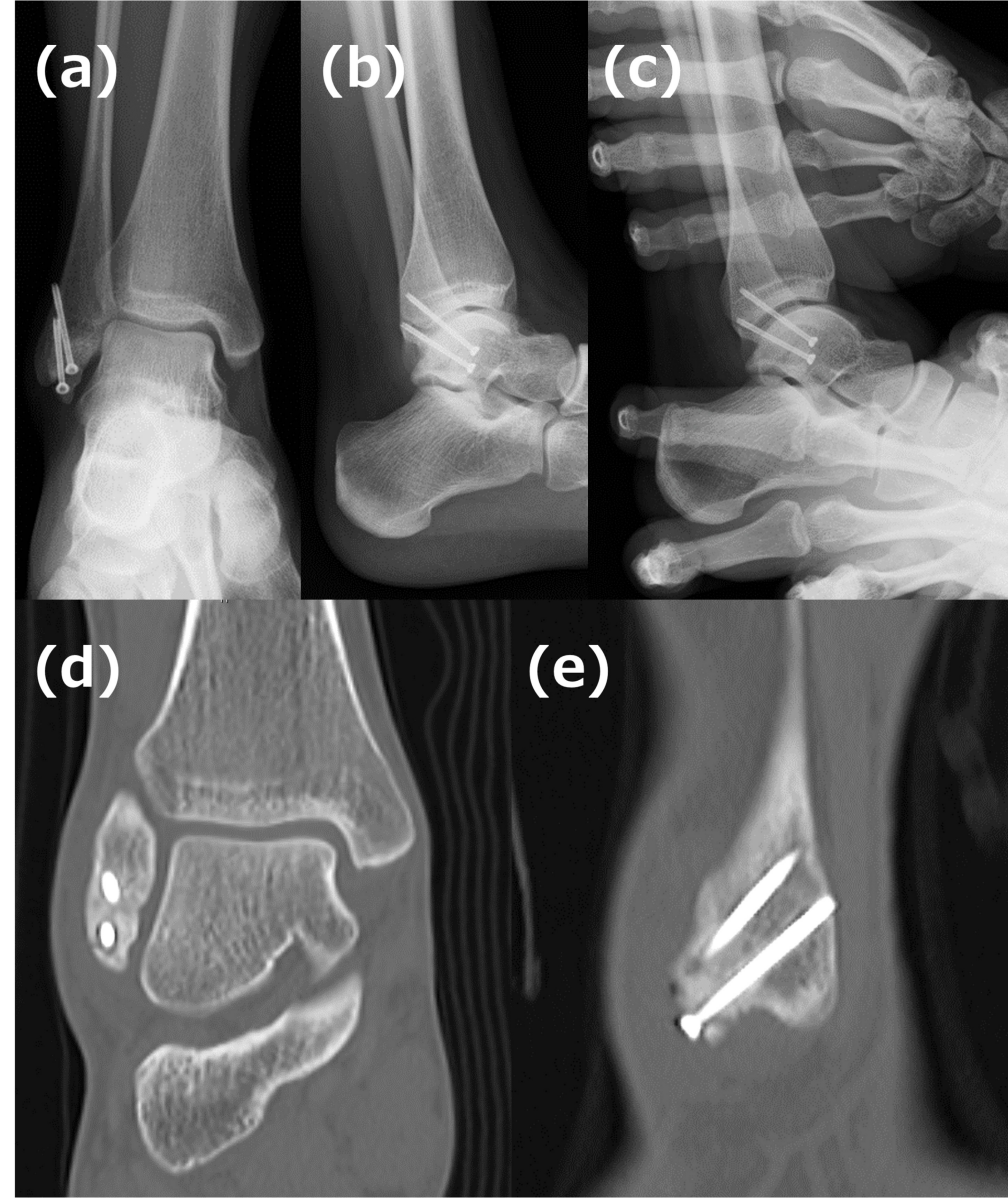
Postoperative imaging at one year. Plain radiographs: Anteroposterior view (a), lateral view (b), and anterior drawer test (c). The anterior drawer test indicates improved stability compared to preoperative findings. CT images: Coronal view (d) and sagittal view (e), illustrating successful bone union.

Postoperatively, the patient was immobilized and advised to remain non-weight-bearing for five weeks, followed by progressive weight-bearing and range-of-motion exercises. Full weight-bearing was permitted at seven weeks. At one year, she reported complete symptom resolution, with no pain or instability. Radiographs confirmed bone union and improved ankle joint stability, and the patient demonstrated a full range of motion (Figure 3).

The Japanese Society for Surgery of the Foot score improved from 93/100 preoperatively to 95/100 postoperatively, indicating improved functional capacity in daily activities [10,11].

## Discussion

While often incidental, os subfibulare can pose significant clinical challenges when symptomatic. Its pathophysiology frequently involves accessory ossification or ATFL avulsion fractures, leading to chronic instability [1-3]. The decision to pursue surgical intervention depends on fragment size, ligament integrity, and instability. Although fragment excision is a viable option for smaller ossicles, it may exacerbate instability in cases with larger fragments or intact ligament attachments, often necessitating concurrent ligament reconstruction [12].

In this case, the decision to fix the 14 mm fragment was guided by its size and the intact attachment of the ATFL, which fixation preserved. Prior literature supports fixation as a means to restore joint stability and maintain ligamentous integrity, particularly in larger fragments [8,12]. Notably, studies underscore the importance of preserving ligament attachments to optimize outcomes. As employed in this case, CCS fixation provided a stable construct with minimal disruption to surrounding structures, aligning with current evidence advocating for fixation in similar scenarios.

However, the optimal fragment size threshold for fixation versus excision remains undefined. While larger fragments are generally associated with better outcomes following fixation, precise criteria for determining surgical approach are lacking. This highlights the need for further research to establish standardized guidelines based on fragment size, ligament status, and patient-specific factors such as activity level and age.

Reports of fixation techniques for os subfibulare remain limited. While methods such as tension band wiring, headless compression screws, and CCS fixation have been described, reports remain scarce [8,13-15]. In this case, we used two 2.2 mm CCSs, which provided sufficient stability, prevented rotational displacement, and were expected to promote bone union. Larger screws can offer greater fixation strength per screw due to their increased diameter; however, their use also presents potential disadvantages. Larger screws may reduce the contact surface area between bone fragments, potentially leading to a lower rate of bone union. Additionally, they may limit the number of screws that can be inserted and increase the risk of fragment splitting, potentially compromising overall fixation stability in smaller bone fragments. Although studies on medial malleolus fractures have reported that using a single screw increases the likelihood of non-anatomical reduction without affecting nonunion or reoperation rates, the optimal screw diameter and number remain subjects of debate [16]. This underscores the need for further investigation into these parameters in the context of os subfibulare fixation to establish evidence-based surgical strategies. Comparative analyses of fixation methods are essential to clarify their relative advantages, limitations, and the long-term durability of outcomes. However, the short-term follow-up limits the generalizability of these findings. Future research should evaluate the long-term durability of outcomes and compare different fixation methods to establish evidence-based surgical strategies.

This case contributes to the growing body of evidence advocating for fixation as a reliable approach in managing symptomatic large os subfibulare. CCSs provided stable fixation with minimal surgical trauma, aligning with outcomes reported in prior studies. The procedure effectively restored ankle stability and resolved symptoms without necessitating adjunctive ligament reconstruction, reinforcing its role as a first-line surgical option for larger fragments. Further exploration into the biomechanical properties of fixation techniques and their clinical implications could pave the way for improved management strategies.

## Conclusions

Cannulated cancellous screw fixation is a reliable and effective surgical approach for managing symptomatic os subfibulare, particularly in cases involving large fragments with intact ligament attachments. This technique restores ankle stability, alleviates pain, and ensures favorable short-term functional outcomes. However, controversies remain regarding fragment size thresholds, optimal screw parameters, and long-term outcomes. Future studies should aim to refine surgical indications, evaluate the biomechanical advantages of different fixation techniques, and establish standardized treatment protocols to optimize care for patients with symptomatic os subfibulare.

## References

[1] Griffiths JD, Menelaus MB (1987). Symptomatic ossicles of the lateral malleolus in children. J Bone Joint Surg Br.

[2] Berg EE (1991). The symptomatic os subfibulare. J Bone Joint Surg Am.

[3] Betz RR, Cooperman DR, Wopperer JM (1990). Late sequelae of septic arthritis of the hip in infancy and childhood. J Pediatr Orthop.

[4] Bowlus TH, Korman SF, Desilvio M, Climo R (1980). Accessory os fibulare avulsion secondary to the inversion ankle injury. J Am Podiatry Assoc.

[5] Powell HDW (1961). Extra centre of ossification for the medial malleolus in children: incidence and significance. J Bone Joint Surg Br.

[6] Choi WJ, Lee JW, Han SH, Kim BS, Lee SK (2008). Chronic lateral ankle instability: the effect of intra-articular lesions on clinical outcome. Am J Sports Med.

[7] Strauss JE, Forsberg JA, Lippert FG III (2007). Chronic lateral ankle instability and associated conditions: a rationale for treatment. Foot Ankle Int.

[8] Kose O, Kilicaslan OF, Guler F, Aktan C (2015). Intraarticular entrapment of os subfibulare following a severe inversion injury of the ankle: a case report. Arch Trauma Res.

[9] Hong CC, Tan KJ, Calder J (2024). Chronic lateral ankle ligament instability - current evidence and recent management advances. J Clin Orthop Trauma.

[10] Niki H, Aoki H, Inokuchi S (2005). Development and reliability of a standard rating system for outcome measurement of foot and ankle disorders I: development of standard rating system. J Orthop Sci.

[11] Niki H, Aoki H, Inokuchi S (2005). Development and reliability of a standard rating system for outcome measurement of foot and ankle disorders II: interclinician and intraclinician reliability and validity of the newly established standard rating scales and Japanese Orthopaedic Association rating scale. J Orthop Sci.

[12] Kim BS, Choi WJ, Kim YS, Lee JW (2010). The effect of an ossicle of the lateral malleolus on ligament reconstruction of chronic lateral ankle instability. Foot Ankle Int.

[13] Lui TH, Wan YT (2019). Arthroscopic stabilization of unstable os subfibulare. Arthrosc Tech.

[14] Faraj AA, Alcelik I (2003). Recurrent ankle sprains secondary to nonunion of a lateral malleolus fracture. J Foot Ankle Surg.

[15] El Ashry SR, El Gamal TA, Platt SR (2017). Atypical chronic ankle instability in a pediatric population secondary to distal fibula avulsion fracture nonunion. J Foot Ankle Surg.

[16] Aamir J, Caldwell R, Long S (2024). A retrospective case series of single-screw vs dual-screw fixation for treatment of medial malleolus fractures. Foot Ankle Orthop.

